# A comprehensive systematic review and network meta-analysis: the role of anti-angiogenic agents in advanced epithelial ovarian cancer

**DOI:** 10.1038/s41598-022-07731-1

**Published:** 2022-03-09

**Authors:** Aya El Helali, Charlene H. L. Wong, Horace C. W. Choi, Wendy W. L. Chan, Naomi Dickson, Steven W. K. Siu, Karen K. Chan, Hextan Y. S. Ngan, Roger K. C. Ngan, Richard D. Kennedy

**Affiliations:** 1 Department of Clinical Oncology, Li Ka Shing Faculty of Medicine, The University of Hong Kong, Queen Mary Hospital, 1/F Professorial Block, 102 Pokfulam Road, Hong Kong SAR, People’s Republic of China; 2 The Patrick G Johnston Centre for Cancer Research, Department of Medical Oncology, Queen’s University Belfast, Belfast, Northern Ireland, UK; 3 Division of Epidemiology and Biostatistics, The School of Public Health, Division of Epidemiology and Biostatistics, Li Ka Shing Faculty of Medicine, The University of Hong Kong, Hong Kong SAR, People’s Republic of China; 4 Department of Obstetrics and Gynaecology, Queen Mary Hospital, The University of Hong Kong, Hong Kong SAR, People’s Republic of China

**Keywords:** Medical research, Oncology

## Abstract

The efficacy of anti-angiogenic agents (AAAs) in epithelial ovarian cancer (EOC) remains unclear. Therefore, we conducted a systematic review and network meta-analysis (NMA) to synthesize evidence of their comparative effectiveness for improving overall survival (OS) among EOC patients. We searched six databases for randomized controlled trials (RCTs) from their inception to February 2021. We performed an NMA with hazard ratios (HRs) and 95%-confidence intervals (CIs) to evaluate comparative effectiveness among different AAAs in chemotherapy-naïve and recurrent EOC. P-score was used to provide an effectiveness hierarchy ranking. Sensitivity NMA was carried out by focusing on studies that reported high-risk chemotherapy-naïve, platinum-resistant, and platinum-sensitive EOC. The primary outcome was OS. We identified 23 RCTs that assessed the effectiveness of AAAs. In recurrent EOC, concurrent use of trebananib (10 mg/kg) with chemotherapy was likely to be the best option (P-score: 0.88, HR 1.67, 95% CI 0.94; 2.94). The NMA indicated that bevacizumab plus chemotherapy followed by maintenance bevacizumab (P-score: 0.99) and pazopanib combined with chemotherapy (P-score: 0.79) both had the highest probability of being the best intervention for improving OS in high-risk chemotherapy-naïve and platinum-resistant EOC, respectively. AAAs may not play a significant clinical role in non-high-risk chemotherapy-naïve and platinum-sensitive EOC.

## Introduction

Epithelial ovarian cancer (EOC) is the 8th most common female reproductive cancer-related cause of death^1^. The majority of tumors relapse following platinum-based chemotherapy, which is associated with poor outcomes. Despite significant improvements in the management of relapsed EOC, the treatment options remain limited. Novel strategies have entered the clinic to manage recurrent EOC, including the use of anti-angiogenic therapies and PARP inhibitiors^2–6^.

Approximately 70% of patients with EOC will eventually relapse following first-line systemic chemotherapy. The fifth Gynecologic Cancer Intergroup (GCIG) recommended re-categorizing platinum sensitivity based on platinum-free interval (PFI) duration (< 1 month, 1–6 months, 6–12 months, and > 12 months). These subsets corresponded to the previously widely used categories of platinum-refractory, platinum-resistant, partially platinum-sensitive, and fully platinum-sensitive, attempting to standardize second-line therapy^7,8^. Patients with a PFI < 6 months have fewer alternative treatment options and consequently poorer survival outcomes^9,10^.

Anti-angiogenic agents have attained regulatory approval to manage a diverse spectrum of solid cancers. Angiogenesis is a crucial pathological^11–16^ hallmark of EOC, and anti-angiogenic agents have dominated the field of drug development in EOC, particularly in the setting of recurrent disease. In EOC, bevacizumab is the only anti-angiogenic agent approved by the Food and Drug Administration (FDA) and European Medicines Agency (EMA). The FDA approved bevacizumab in platinum-resistant EOC^2^ and platinum-sensitive EOC^3,17^ combined with chemotherapy. Additionally, the EMA has approved the clinical use of bevacizumab in EOC irrespective of disease state: chemotherapy-naïve^18,19^; platinum-sensitive^3^, and platinum-resistant^2^ disease.

The approval of other anti-angiogenic agents by the regulatory authorities has been more challenging. The FDA and EMA assigned orphan designation for trebananib and cediranib combined with chemotherapy in the management of recurrent EOC. The National Comprehensive Cancer Network (NCCN)^20^ and the ESMO-ESGO Ovarian Cancer Consensus Conference Working Group^21^ advocate for the clinical use of bevacizumab in both the chemotherapy-naïve setting and the recurrent disease setting. However, the National Institute for Health and Care Excellence (NICE)^22^ does not support the clinical use of bevacizumab in advanced EOC.

Several meta-analyses have been published to shed light on this clinical knowledge gap. Unfortunately, these studies further demonstrate the clinical uncertainty and inconsistent reporting regarding the clinical efficacy of anti-angiogenic agents in the EOC disease state spectrum^23–32^ (Supplementary Table 2). We, therefore, conducted a systematic review and network meta-analysis (NMA) to synthesize evidence for the comparative effectiveness of different anti-angiogenic agents for improving overall survival (OS) among EOC patients.

## Method

We performed a systematic review and NMA of the curated literature according to the Preferred Reporting Items for Systematic Reviews and Meta-Analyses (PRISMA) extension statement for NMA^33^. A prospective protocol was registered in PROSPERO (registration: CRD42021240133)^34^.

### Eligibility criteria

To be eligible for inclusion in this review, a randomized controlled trial (RCT) should be published in English and satisfy the following criteria: Patient, Intervention, comparison, and Outcome (PICO). Patients with either locally advanced or recurrent EOC were included. In addition, as defined in the current evidence-based guidelines, anti-angiogenic agents, compared with placebo or any type of standard of care chemotherapy, were considered eligible. Included RCTs should report hazard ratio (HR) and 95% confidence intervals (CI) for the outcomes of overall survival (OS) or progression-free survival (PFS) in both intervention and control groups. The primary outcome of this systematic review was OS, while the secondary outcome was PFS.

### Literature search

A systematic search was undertaken to identify RCTs by using the following databases from inception to February 2021: EMBASE; PubMed; Ovid Medline; Cochrane Central Register of Controlled Trials (CCTR); Cochrane Database of Systematic Reviews (CDSR); and ClinicalTrials.gov (www.clinicaltrials.gov), ASCO and ESMO abstract database. Furthermore, we searched references of relevant articles retrieved from the electronic search for additional citations. No restrictions on publication status were imposed. The key search terms used were "ovarian cancer", "ovarian neoplasm” [MESH Term], "anti angiogenic agents", "angiogenesis inhibitors", "Bevacizumab", "Nintedanib", "Pazopanib", "Cediranib", "Trebananib", "Sorafenib", "platinum sensitive ovarian cancer", "platinum resistant ovarian cancer", "*VEGF*", "*VEGFR*", "*PDGF*", "*PDGFR*", "*FGF*", "*FGFR*", "*TIE*", "*RET*", "*AXL*", "*FLT*" and "*FLT-3*".

### Study selection, data extraction, and quality assessment

After a comprehensive literature search, we used reference management software (EndNote) to identify and remove potential duplicate RCTs. Two review authors (AE, WLC) independently screened titles and abstracts of the retrieved RCTs and assessed full texts for eligibility. Discrepancies were resolved through consensus; unsolved discrepancies were settled via consulting a third review author (ND). In the event of duplicate publications, priority was given to the publication reporting the most extended follow‐up associated with our primary and secondary outcomes. To be included in the NMA, RCTs should share a common comparator that serves as a bridge for indirect comparison of different anti-angiogenic agents.

Two review authors (AE, HC) collected the following information from each included RCT using an established data abstraction method: year of publication, country, disease settings, number of patients randomized, patient demographics, details of interventions and comparators, results of prespecified outcomes, and study funding sources. Data extracted was further independently evaluated by one author (CW).

Two review authors (AE, WLC) assessed the risk of bias of each eligible RCT independently using the Cochrane risk of bias tool 2^35^. The following domains were assessed: bias arising from the randomization process; bias due to deviations from the intended interventions; bias due to missing outcome data; bias in the measurement of the outcome; and bias in selecting the reported results^36^. The overall risk of bias of each trial was judged as low, some concerns, or high risk of bias^36^. Disagreements were resolved through consensus; unresolved disagreements were settled via consulting a third review author (HC).

### Data analysis

#### NMA

A NMA offers methods to visualize and interpret a broader picture of current evidence and assesses the comparative effectiveness among various interventions^36^. It provides indirect evidence (estimates between various interventions via common comparators) when direct evidence (head-to-head estimates of various interventions) is not available^37^. Therefore, we conducted an indirect comparison between different interventions on the primary and secondary outcomes. In this systematic review, an NMA was performed to investigate the "relatively most" effective anti-angiogenic agent for improving OS and PFS among EOC patients in two disease settings, namely (i) chemotherapy naïve and (ii) recurrent EOC.

The NMA was conducted using the "netmeta" package in R (version 4.1.1)^38,39^. This package is based on a novel approach for frequentist NMA that follows the graph-theoretical methodology^38^. It accounts for the correlated treatment effects in multi-arm trials by reweighting all comparisons of each multi-arm study^39^. Furthermore, the frequentist NMA model calculates the probability of significance for accepting or rejecting the research hypothesis when the data is repeated infinitely based on a general statistical theory^40^. Therefore, results produced by the frequentist approach would be easy to interpret^41^. The frequentist NMA also considers heterogeneity between studies and inconsistency between study designs. It facilitates the incorporation of heterogeneity and inconsistency in the effect estimation^42^. The random-effects model was selected considering the between-study variation^41^. Thus, the effect estimates would be more realistic^43^. A network plot was generated for each disease setting to show all interventions included in the NMA^38,39^. Comparative effectiveness results of all possible comparisons were summarized with a HR and 95% CI^38,39^. P-score was used to provide an effectiveness hierarchy ranking^38,39^. A higher P-score represents superior performance^44^.

Given the potential impact of anti-angiogenic agents in the (i) chemotherapy naïve high-risk group^45^, ii) platinum-resistant and (iii) platinum-sensitive groups in recurrent EOC setting, sensitivity analyses were conducted by only including studies that explored the effectiveness of anti-angiogenic agents on the primary and secondary outcomes in these three group of patients within the corresponding disease settings. High-risk disease was defined as FIGO stage IV or inoperable stage or sub-optimally (> 1 cm residual disease) resected FIGO stage III^46^.

The validity of the NMA relied on the assumption of transitivity, which required that different sets of studies included in the analysis were similar in population, study designs, and outcomes, apart from the intervention comparison being made^47,48^. Transitivity should hold for every possible indirect comparison^49^. The statistical manifestation of transitivity was the consistency of direct and indirect evidence on the same comparison^41,50^. In a valid NMA, consistency should hold in every loop of evidence within the network^49^. Therefore, the separating indirect from direct evidence (SIDE) approach using the back-calculation method was performed to evaluate consistency in a loop of evidence within the network^51^. The inconsistency with the p-value less than 0.05 was considered a significant concern.

#### Assessment of publication bias

Publication bias on the primary outcome in each disease setting was assessed via a comparison-adjusted funnel plot produced by R (version 4.1.1)^38,39^ when at least ten studies were included in the NMA^35^.

## Results

### Study selection and characteristics of included RCTs

A total of 2363 publications were identified through the initial literature search, and 1563 studies remained after duplications were excluded. Following the title and abstract screening process, 1392 publications were removed because they did not meet the study's hypothesis or were abstracts of full-text publications included in the eligible articles review. 171 potentially relevant articles were identified in the comprehensive review. Following this process, 23 multicentre phase II–III RCTs were analyzed in this NMA. Details of literature search and selection for RCTs are presented in Supplementary Fig. 1.

Characteristics of the 23 included RCTs are summarized in Supplementary Table 1. They included 11,560 patients, with sample sizes varying from 84 to 1873 patients. Four RCTs were conducted in the chemotherapy naïve setting, three in the first-line maintenance setting, and 16 in the recurrent EOC setting. Eight different anti-angiogenic agents administered concurrently with chemotherapy, with or without maintenance treatment, were evaluated in all included trials. Standard of care chemotherapy was reported as a control intervention in 19 trials. All RCTs assessed OS and PFS, with the exception of the Duska et al.^52^ and the East Asian Study^53^ trials reported PFS only.

### Risk of bias among included RCTs

Regarding the overall risk of bias, 12 RCTs were rated as low (52.2%). The remaining 11 RCTs were rated as having some concerns (47.8%). All the included RCTs had a low risk of bias in the two domains: (i) bias due to missing outcome data and (ii) bias in the selection of the reported result. Approximately 20% and 35% of RCTs had some concerns on bias due to deviations from the intended interventions and bias in the outcome measurement, respectively. Only 8.7% had some concerns on bias arising from the randomization process. Details of risk of bias assessment on each domain are presented in Supplementary Tables 3 and 4.

### NMA results

#### Chemotherapy naïve setting

**Figure 1:**
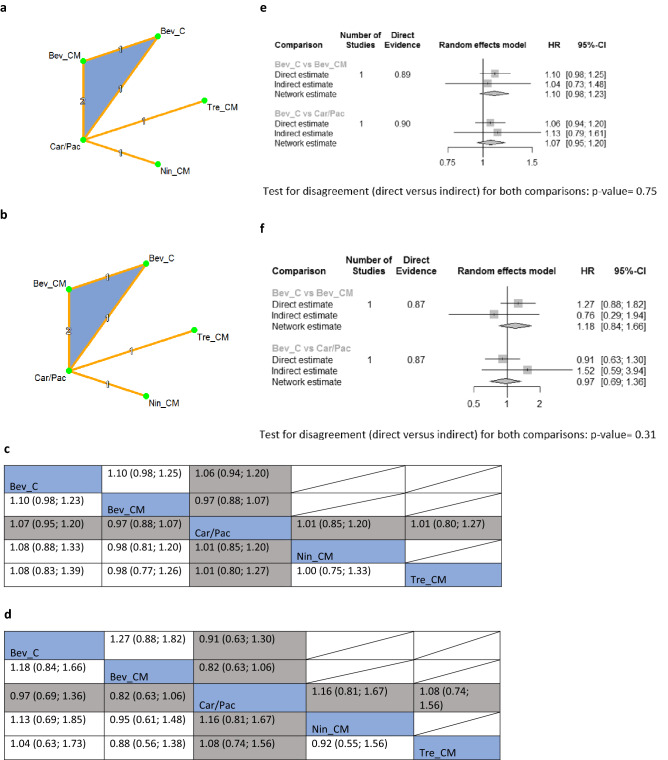
(**a**) Network plot of comparisons among different anti-angiogenic agents and standard of care chemotherapy (i.e., Carboplatin/Paclitaxel) for overall survival in a chemotherapy naïve setting. (**b**) Network plot of comparisons among different anti-angiogenic agents and standard of care chemotherapy (i.e., Carboplatin/Paclitaxel) for progression-free survival in chemotherapy naïve setting. Keys for (**a**, **b**): *Bev_C* Bevacizumab (concurrent), *Bev_CM* Bevacizumab (concurrent + maintenance), *Car/Pac* Carboplatin/Paclitaxel, *Nin_CM* Nintedanib (concurrent + maintenance), *Tre_CM* Trebananib (concurrent + maintenance). Notes for (**a**, **b**): Nodes represent the interventions, lines connecting nodes represent direct comparisons between pairs of interventions, the number stated on the lines represents the number of studies involved in the comparisons. (**c**) Comparative effectiveness of different anti-angiogenic agents and standard of care chemotherapy (i.e., Carboplatin/Paclitaxel) for overall survival in chemotherapy naïve setting. (**d**) Comparative effectiveness of different anti-angiogenic agents and standard of care chemotherapy (i.e., Carboplatin/Paclitaxel) for progression-free survival in chemotherapy naïve setting. Keys for (**c**, **d**): *Bev_C* Bevacizumab (concurrent), *Bev_CM* Bevacizumab (concurrent + maintenance), *Car/Pac* Carboplatin/Paclitaxel, *Nin_CM* Nintedanib (concurrent + maintenance), *Tre_CM* Trebananib (concurrent + maintenance). Notes for (**c**, **d**): The upper triangle of the matrix displays only the pooled effect sizes of the direct comparisons available in the network. Fields that remain empty in the upper triangle mean that the direct evidence for the comparison is not available. The lower triangle shows the estimated effect sizes for each comparison even when only indirect evidence is available. In the lower triangle of the matrix, values in each cell represent the hazard ratio (HR and 95% confidence interval) of the intervention at the top, compared to the comparator on the left. When HR < 1, prefers the column intervention, indicating that the column intervention is more effective than the row intervention on reducing overall survival. When HR > 1, prefers the row intervention. Significant results are in bold and underlined for both upper and lower triangles. (**e**) Results based on the Separating Indirect from Direct Evidence (SIDE) approach to evaluating inconsistency in the network meta-analysis for overall survival in a chemotherapy naïve setting. (**f**) Results based on the Separating Indirect from Direct Evidence (SIDE) approach to evaluating inconsistency in the network meta-analysis for progression-free survival in a chemotherapy naïve setting. Keys for (**e**, **f**) *Bev_C* Bevacizumab (concurrent), *Bev_CM* Bevacizumab (concurrent + maintenance), *Car/Pac* Carboplatin/Paclitaxel.

To demonstrate the impact on OS and PFS, each network included one three-arm trial and three two-arm trials (Fig. 1a,b, respectively). The standard of care chemotherapy in these networks was carboplatin combined with paclitaxel (Car/Pac). The NMA results suggested that the concurrent use of anti-angiogenic agents with standard of care chemotherapy with or without maintenance treatment resulted in no significant difference in OS and PFS outcomes (Fig. 1c,d). Effectiveness hierarchy ranking results of the interventions are shown in Supplementary Fig. 2. As the inconsistency p-values for the two comparisons in OS and PFS were 0.75 and 0.31, respectively (Fig. 1e,f), there was no significant inconsistency in these two NMA.

#### High-risk group

**Figure 2:**
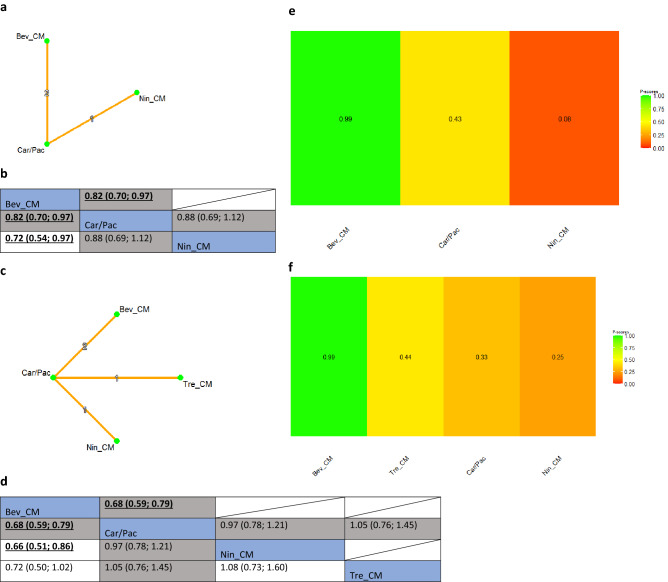
(**a**) Network plot of comparisons among different anti-angiogenic agents and standard of care chemotherapy (i.e., Carboplatin/Paclitaxel) for overall survival for the high-risk group in the chemotherapy naïve setting. Key for (**a**): *Bev_CM* Bevacizumab (concurrent + maintenance), *Car/Pac* Carboplatin/Paclitaxel, *Nin_CM* Nintedanib (concurrent + maintenance). Note for (**a**): Nodes represent the interventions, lines connecting nodes represent direct comparisons between pairs of interventions, the number stated on the lines represents the number of studies involved in the comparisons. (**b**) Comparative effectiveness of different anti-angiogenic agents and standard of care chemotherapy (i.e., Carboplatin/Paclitaxel) for overall survival for the high-risk group in chemotherapy naïve setting. Key for (**b**): *Bev_CM* Bevacizumab (concurrent + maintenance), *Car/Pac* Carboplatin/Paclitaxel, *Nin_CM* Nintedanib (concurrent + maintenance). Note for (**b**): The upper triangle of the matrix displays only the pooled effect sizes of the direct comparisons available in the network. Fields that remain empty in the upper triangle mean that the direct evidence for the comparison is not available. The lower triangle shows the estimated effect sizes for each comparison even when only indirect evidence is available. In the lower triangle of the matrix, values in each cell represent the hazard ratio (HR and 95% confidence interval) of the intervention at the top, compared to the comparator on the left. When HR < 1, prefers the column intervention, indicating that the column intervention is more effective than the row intervention on reducing overall survival. When HR > 1, prefers the row intervention. Significant results are in bold and underlined for both upper and lower triangles. (**c**) Network plot of comparisons among different anti-angiogenic agents and standard of care chemotherapy (i.e., Carboplatin/Paclitaxel) for progression-free survival for the high-risk group in chemotherapy naïve setting. Key for (**c**): *Bev_CM* Bevacizumab (concurrent + maintenance), *Car/Pac* Carboplatin/Paclitaxel, *Nin_CM* Nintedanib (concurrent + maintenance), *Tre_CM* Trebananib (concurrent + maintenance). Note for (**c**): Nodes represent the interventions, lines connecting nodes represent direct comparisons between pairs of interventions, the number stated on the lines represents the number of studies involved in the comparisons. (**d**) Comparative effectiveness of different anti-angiogenic agents and standard of care chemotherapy (i.e., Carboplatin/Paclitaxel) for progression-free survival for the high-risk group in chemotherapy naïve setting. Key for (**d**): *Bev_CM* Bevacizumab (concurrent + maintenance), *Car/Pac* Carboplatin/Paclitaxel, *Nin_CM* Nintedanib (concurrent + maintenance), *Tre_CM* Trebananib (concurrent + maintenance). Note for (**d**): The upper triangle of the matrix displays only the pooled effect sizes of the direct comparisons available in the network. Fields that remain empty in the upper triangle mean that the direct evidence for the comparison is not available. The lower triangle shows the estimated effect sizes for each comparison even when only indirect evidence is available. In the lower triangle of the matrix, values in each cell represent the hazard ratio (HR and 95% confidence interval) of the intervention at the top, compared to the comparator on the left. When HR < 1, prefers the column intervention, indicating that the column intervention is more effective than the row intervention on reducing overall survival. When HR > 1, prefers the row intervention. Significant results are in bold and underlined for both upper and lower triangles. (**e**) Comparative effectiveness of different anti-angiogenic agents and standard of care chemotherapy (i.e., Carboplatin/Paclitaxel) for the high-risk group in chemotherapy naïve setting: P-scores for overall survival. Key for (**e**): *Bev_CM* Bevacizumab (concurrent + maintenance), *Car/Pac* Carboplatin/Paclitaxel, *Nin_CM* Nintedanib (concurrent + maintenance). Note for (**e**): The higher the P-scores, the higher likelihood that an intervention is in the top rank or one of the top ranks. (**f**) Comparative effectiveness of different anti-angiogenic agents and standard of care chemotherapy (i.e., Carboplatin/Paclitaxel) for the high-risk group in chemotherapy naïve setting: P-scores for progression-free survival. Key for (**b**): *Bev_CM* Bevacizumab (concurrent + maintenance), *Car/Pac* Carboplatin/Paclitaxel, *Nin_CM* Nintedanib (concurrent + maintenance), *Tre_CM* Trebananib (concurrent + maintenance). Notes for (**f**): The higher the P-scores, the higher likelihood that an intervention is in the top rank or one of the top ranks.

There were four RCTs in the chemotherapy naïve high-risk group. The ICON7 trial defined high-risk disease as FIGO stage IV, inoperable, or sub-optimally (> 1 cm residual disease) resected FIGO stage III^46^. These criteria were shared with the TRINOVA-3^54^, GOG-0218 trials ^55^, and the AGO-OVAR 12^56^. Results of the NMA sensitivity analysis supported the use of bevacizumab, administered concurrently with chemotherapy followed by maintenance treatment (Bev_CM). In the chemotherapy naïve high-risk group, bevacizumab was associated with an improved OS (P-score: 0.99, NMA estimate of Bev_CM versus Car/Pac: HR 0.82, 95% CI 0.70; 0.97) (Fig. 2a,b) and PFS (P-score: 0.99, NMA estimate of Bev_CM versus Car/Pac: HR 0.68, 95% CI 0.59; 0.79) (Fig. 2c,d). Bevacizumab was likely to be the best treatment option for both outcomes for this group of patients. Effectiveness hierarchy ranking results of the interventions for OS and PFS are shown in Fig. 2e and f, respectively.

#### Recurrent EOC setting

**Figure 3:**
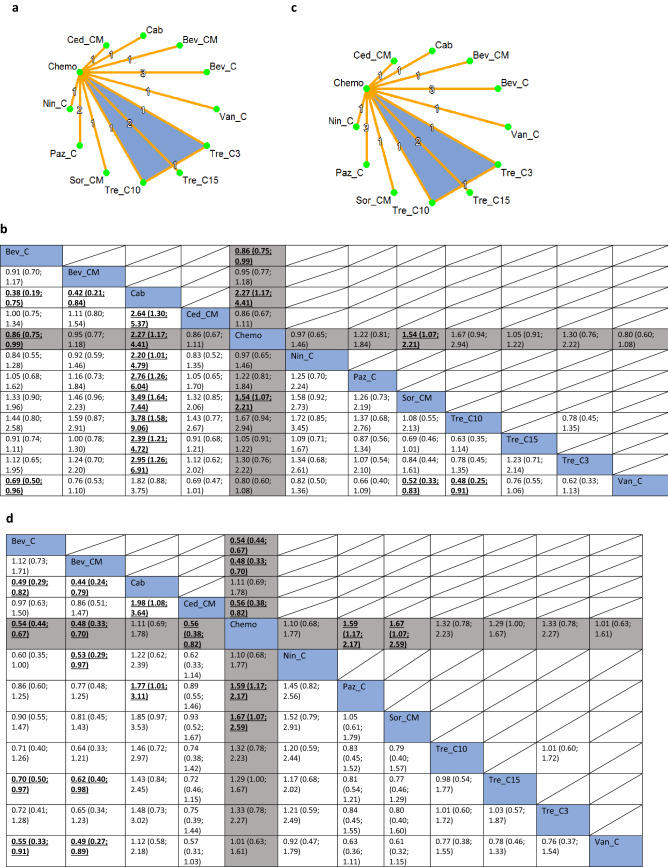

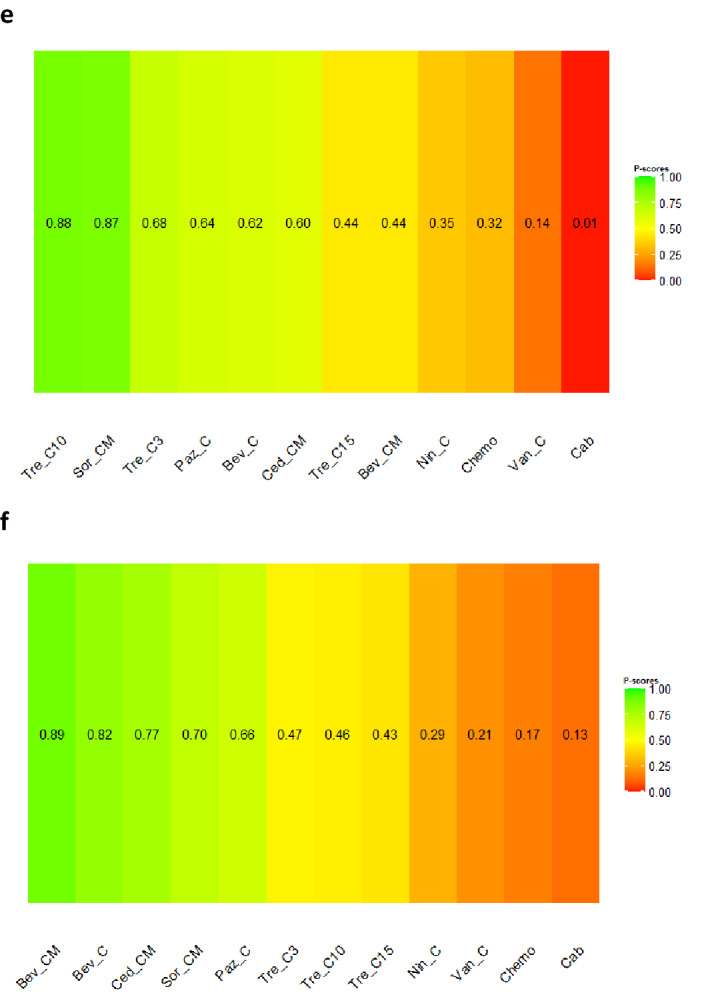
(**a**) Network plot of comparisons among different anti-angiogenic agents and standard of care chemotherapy for overall survival in recurrent EOC setting. Key for (**a**): *Bev_C* Bevacizumab (concurrent), *Bev_CM* Bevacizumab (concurrent + maintenance), *Cab* Cabozantinib, *Ced_CM* Cediranib (concurrent + maintenance), *Chemo* Standard of care chemotherapy, *Nin_C* Nintedanib (concurrent), *Paz_C* Pazopanib (concurrent), *Sor_CM* Sorafenib (concurrent + maintenance), *Tre_C3* Trebananib (3 mg/kg) (concurrent), *Tre_C10* Trebananib (10 mg/kg) (concurrent), *Tre_C15* Trebananib (15 mg/kg) (concurrent); Van_C, Vandetanib (concurrent). Note for (**a**): Nodes represent the interventions, lines connecting nodes represent direct comparisons between pairs of interventions, number stated on the lines represent the number of studies involved in the comparisons. (**b**) Comparative effectiveness of different anti-angiogenic agents and standard of care chemotherapy for overall survival in recurrent EOC setting. Key for (**b**): *Bev_C* Bevacizumab (concurrent), *Bev_CM* Bevacizumab (concurrent + maintenance), *Cab* Cabozantinib, *Ced_CM* Cediranib (concurrent + maintenance), *Chemo* Standard of care chemotherapy, *Nin_C* Nintedanib (concurrent), *Paz_C* Pazopanib (concurrent), *Sor_CM* Sorafenib (concurrent + maintenance), *Tre_C3* Trebananib (3 mg/kg) (concurrent), *Tre_C10* Trebananib (10 mg/kg) (concurrent), *Tre_C15* Trebananib (15 mg/kg) (concurrent), *Van_C* Vandetanib (concurrent). Note for (**b**): The upper triangle of the matrix displays only the pooled effect sizes of the direct comparisons available in the network. Fields that remain empty in the upper triangle mean that the direct evidence for the comparison is not available. The lower triangle shows the estimated effect sizes for each comparison even when only indirect evidence is available. In the lower triangle of the matrix, values in each cell represent the hazard ratio (HR and 95% confidence interval) of the intervention at the top, compared to the comparator on the left. When HR < 1, prefers the column intervention, indicating that the column intervention is more effective than the row intervention on reducing overall survival. When HR > 1, prefers the row intervention. Significant results are in bold and underlined for both upper and lower triangles. (**c**) Network plot of comparisons among different anti-angiogenic agents and standard of care chemotherapy for progression-free survival in recurrent EOC setting. Key for (**b**): *Bev_C* Bevacizumab (concurrent), *Bev_CM* Bevacizumab (concurrent + maintenance), *Cab* Cabozantinib, *Ced_CM* Cediranib (concurrent + maintenance), *Chemo* Standard of care chemotherapy, *Nin_C* Nintedanib (concurrent), *Paz_C* Pazopanib (concurrent), *Sor_CM* Sorafenib (concurrent + maintenance), *Tre_C3* Trebananib (3 mg/kg) (concurrent), *Tre_C10* Trebananib (10 mg/kg) (concurrent), *Tre_C15* Trebananib (15 mg/kg) (concurrent), *Van_C* Vandetanib (concurrent). Note for (**c**): Nodes represent the interventions, lines connecting nodes represent direct comparisons between pairs of interventions, number stated on the lines represent the number of studies involved in the comparisons. (**d**) Comparative effectiveness of different anti-angiogenic agents and standard of care chemotherapy for progression-free survival in recurrent EOC setting. Key for (**d**): *Bev_C* Bevacizumab (concurrent), *Bev_CM* Bevacizumab (concurrent + maintenance), *Cab* Cabozantinib, *Ced_CM* Cediranib (concurrent + maintenance), *Chemo* Standard of care chemotherapy, *Nin_C* Nintedanib (concurrent), *Paz_C* Pazopanib (concurrent), *Sor_CM* Sorafenib (concurrent + maintenance), *Tre_C3* Trebananib (3 mg/kg) (concurrent), *Tre_C10* Trebananib (10 mg/kg) (concurrent), *Tre_C15* Trebananib (15 mg/kg) (concurrent), *Van_C* Vandetanib (concurrent). Note for (**d**): The upper triangle of the matrix displays only the pooled effect sizes of the direct comparisons available in the network. Fields that remain empty in the upper triangle mean that the direct evidence for the comparison is not available. The lower triangle shows the estimated effect sizes for each comparison even when only indirect evidence is available. In the lower triangle of the matrix, values in each cell represent the hazard ratio (HR and 95% confidence interval) of the intervention at the top, compared to the comparator on the left. When HR < 1, prefers the column intervention, indicating that the column intervention is more effective than the row intervention on reducing overall survival. When HR > 1, prefers the row intervention. Significant results are in bold and underlined for both upper and lower triangles. (**e**) Comparative effectiveness of different anti-angiogenic agents and standard of care chemotherapy in recurrent EOC setting: P-scores for overall survival. Key for (**e**): *Bev_C* Bevacizumab (concurrent), *Bev_CM* Bevacizumab (concurrent + maintenance), *Cab* Cabozantinib, *Ced_CM* Cediranib (concurrent + maintenance), *Chemo* Standard of care chemotherapy, *Nin_C* Nintedanib (concurrent), *Paz_C* Pazopanib (concurrent), *Sor_CM* Sorafenib (concurrent + maintenance), *Tre_C3* Trebananib (3 mg/kg) (concurrent), *Tre_C10* Trebananib (10 mg/kg) (concurrent), *Tre_C15* Trebananib (15 mg/kg) (concurrent), *Van_C* Vandetanib (concurrent). Notes for (**e**): The higher the P-scores, the higher likelihood that an intervention is in the top rank or one of the top ranks. (**f**) Comparative effectiveness of different anti-angiogenic agents and standard of care chemotherapy in recurrent EOC setting: P-scores for progression-free survival. Key for (**f**): *Bev_C* Bevacizumab (concurrent), *Bev_CM* Bevacizumab (concurrent + maintenance), *Cab* Cabozantinib, *Ced_CM* Cediranib (concurrent + maintenance), *Chemo* Standard of care chemotherapy, *Nin_C* Nintedanib (concurrent), *Paz_C* Pazopanib (concurrent), *Sor_CM* Sorafenib (concurrent + maintenance), *Tre_C3* Trebananib (3 mg/kg) (concurrent), *Tre_C10* Trebananib (10 mg/kg) (concurrent), *Tre_C15* Trebananib (15 mg/kg) (concurrent); Van_C, Vandetanib (concurrent). Notes for (**f**): The higher the P-scores, the higher likelihood that an intervention is in the top rank or one of the top ranks.

To demonstrate the impact on OS, the network included one three-arm trial and 13 two-arm trials (Fig. 3a). The concurrent use of trebananib (10 mg/kg) and chemotherapy (Tre_C10) was likely to be the best option (P-score: 0.88, NMA estimate of Tre_C10 versus Standard of care chemotherapy (Chemo): HR 0.60, 95% CI 0.34; 1.06), followed by sorafenib combined with chemotherapy and maintenance treatment (Sor_CM) (P-score: 0.87, NMA estimate of Sor_CM versus Chemo: HR 0.65, 95% CI 0.45; 0.93) (Fig. 3b).

To demonstrate the impact on PFS, the network included one three-arm trial and 14 two-arm trials (Fig. 3c). The combination of chemotherapy and maintenance treatment with bevacizumab (Bev_CM) resulted in a significant improvement in PFS when compared to standard of care chemotherapy (HR 0.48, 95% CI 0.33; 0.70) (Fig. 3d). The combination of chemotherapy and maintenance treatment with bevacizumab (Bev_CM) was also likely to be the most effective option (P-score: 0.89), followed by the concurrent use of Bevacizumab and chemotherapy (Bev_C) (P-score: 0.82, NMA estimate of Bev_C versus Chemo: HR 0.54, 95% CI 0.44; 0.67). Effectiveness hierarchy ranking results of the interventions for OS and PFS are shown in Fig. 3e and f, respectively. Nonetheless, consistency could not be evaluated in the two NMAs on OS and PFS as there was no loop of evidence.

#### Platinum resistant group

**Figure 4:**
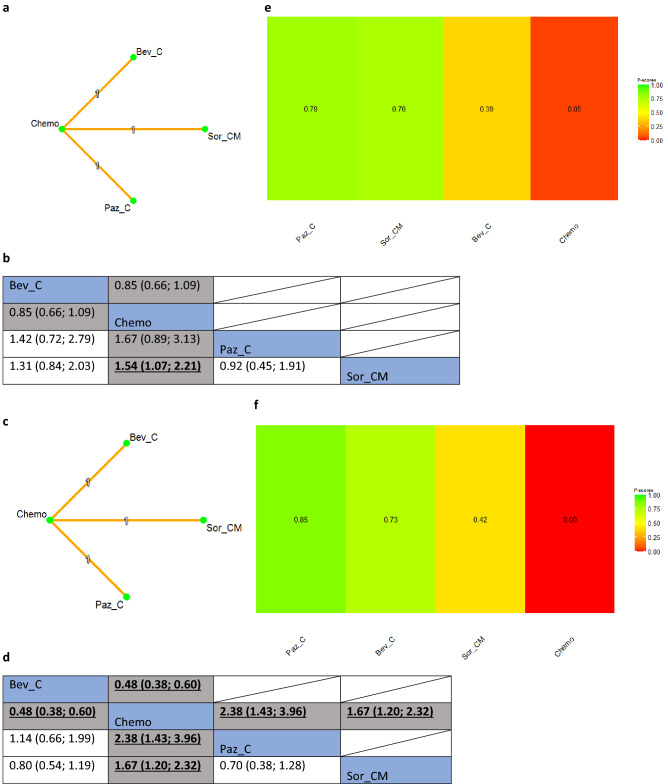
(**a**) Network plot of comparisons among different anti-angiogenic agents and standard of care chemotherapy for overall survival in the platinum-resistant setting. Key for (**a**): *Bev_C* Bevacizumab (concurrent), *Chemo* Standard of care chemotherapy, *Paz_C* Pazopanib (concurrent), *Sor_CM* Sorafenib (concurrent + maintenance). Note for (**a**): Nodes represent the interventions, lines connecting nodes represent direct comparisons between pairs of interventions, the number stated on the lines represents the number of studies involved in the comparisons. (**b**) Comparative effectiveness of different anti-angiogenic agents and standard of care chemotherapy for overall survival in the platinum-resistant setting. Key for (**b**): *Bev_C* Bevacizumab (concurrent), *Chemo* Standard of care chemotherapy, *Paz_C* Pazopanib (concurrent), *Sor_CM* Sorafenib (concurrent + maintenance). Note for (**b**): The upper triangle of the matrix displays only the pooled effect sizes of the direct comparisons available in the network. Fields that remain empty in the upper triangle mean that the direct evidence for the comparison is not available. The lower triangle shows the estimated effect sizes for each comparison even when only indirect evidence is available. In the lower triangle of the matrix, values in each cell represent the hazard ratio (HR and 95% confidence interval) of the intervention at the top, compared to the comparator on the left. When HR < 1, prefers the column intervention, indicating that the column intervention is more effective than the row intervention on reducing overall survival. When HR > 1, prefers the row intervention. Significant results are in bold and underlined for both upper and lower triangles. (**c**) Network plot of comparisons among different anti-angiogenic agents and standard of care chemotherapy for progression-free survival in the platinum-resistant setting. Key for (**c**): *Bev_C* Bevacizumab (concurrent), *Chemo* Standard of care chemotherapy, *Paz_C* Pazopanib (concurrent), *Sor_CM* Sorafenib (concurrent + maintenance). Note for (**c**): Nodes represent the interventions, lines connecting nodes represent direct comparisons between pairs of interventions, the number stated on the lines represents the number of studies involved in the comparisons. (**d**) Comparative effectiveness of different anti-angiogenic agents and standard of care chemotherapy for progression-free survival in the platinum-resistant setting. Key for (**c**): *Bev_C* Bevacizumab (concurrent), *Chemo* Standard of care chemotherapy, *Paz_C* Pazopanib (concurrent), *Sor_CM* Sorafenib (concurrent + maintenance). Note for (**c**): The upper triangle of the matrix displays only the pooled effect sizes of the direct comparisons available in the network. Fields that remain empty in the upper triangle mean that the direct evidence for the comparison is not available. The lower triangle shows the estimated effect sizes for each comparison even when only indirect evidence is available. In the lower triangle of the matrix, values in each cell represent the hazard ratio (HR and 95% confidence interval) of the intervention at the top, compared to the comparator on the left. When HR < 1, prefers the column intervention, indicating that the column intervention is more effective than the row intervention on reducing overall survival. When HR > 1, prefers the row intervention. Significant results are in bold and underlined for both upper and lower triangles. (**e**) Comparative effectiveness of different anti-angiogenic agents and standard of care chemotherapy in platinum-resistant setting: P-scores for overall survival. Key for (**e**): *Bev_C* Bevacizumab (concurrent), *Chemo* Standard of care chemotherapy, *Paz_C* Pazopanib (concurrent), *Sor_CM* Sorafenib (concurrent + maintenance). Notes for (**e**): The higher the P-scores, the higher likelihood that an intervention is in the top rank or one of the top ranks. (**f**) Comparative effectiveness of different anti-angiogenic agents and standard of care chemotherapy in platinum-resistant setting: P-scores for progression-free survival. Key for (**f**): *Bev_C* Bevacizumab (concurrent), *Chemo* Standard of care chemotherapy, *Paz_C* Pazopanib (concurrent), *Sor_CM* Sorafenib (concurrent + maintenance). Notes for (**f**): The higher the P-scores, the higher likelihood that an intervention is in the top rank or one of the top ranks.

There were three RCTs in the platinum-resistant group of recurrent EOC setting. Results of the sensitivity analysis of the NMA showed that the concurrent use of pazopanib and chemotherapy (Paz_C) had the highest probability of being the best intervention for improving OS (P-score: 0.79, NMA estimate of Paz_C versus Chemo: HR 0.60, 95% CI 0.32; 1.12), followed by sorafenib combined with chemotherapy and maintenance treatment (Sor_CM) (P-score: 0.76, NMA estimate of Sor_CM versus Chemo: HR 0.65, 95% CI 0.45; 0.93) (Fig. 4a,b). The former intervention (Paz_C) was also likely to be the most effective intervention for improving PFS (p-score: 0.85, NMA estimate of Paz_C versus Chemo: HR 0.42, 95% CI 0.25; 0.70) (Fig. 4c,d). Effectiveness hierarchy ranking results of the interventions for OS and PFS are shown in Fig. 4e and f, respectively.

#### Platinum sensitive group

**Figure 5:**
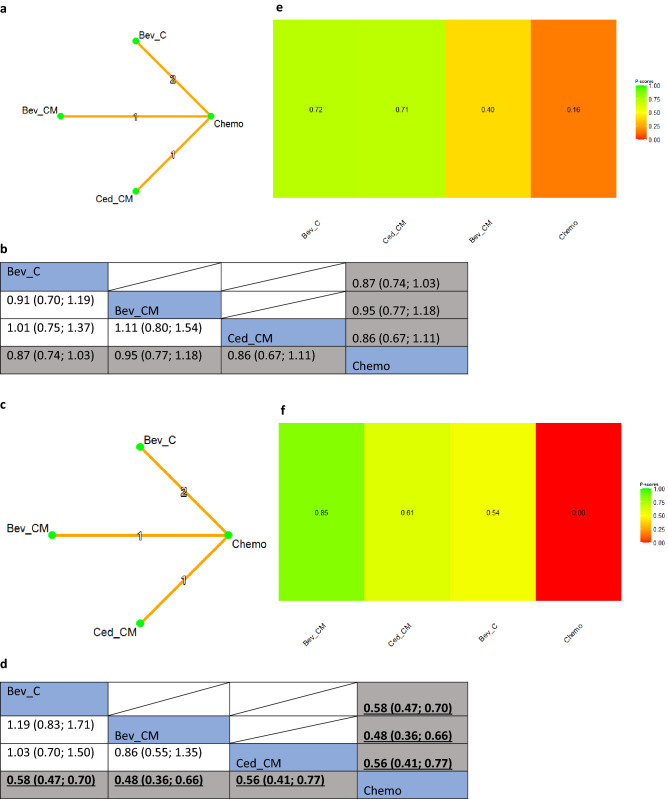
(**a**) Network plot of comparisons among different anti-angiogenic agents and standard of care chemotherapy for overall survival in the platinum-sensitive setting. Key for (**a**): *Bev_C* Bevacizumab (concurrent), *Bev_CM* Bevacizumab (concurrent + maintenance), *Ced_CM* Cediranib (concurrent + maintenance), *Chemo* Standard of care chemotherapy. Note for (**a**): Nodes represent the interventions, lines connecting nodes represent direct comparisons between pairs of interventions, the number stated on the lines represents the number of studies involved in the comparisons. (**b**) Comparative effectiveness of different anti-angiogenic agents and standard of care chemotherapy for overall survival in the platinum-sensitive setting. Key for (**b**): *Bev_C* Bevacizumab (concurrent), *Bev_CM* Bevacizumab (concurrent + maintenance), *Ced_CM* Cediranib (concurrent + maintenance); Chemo, Standard of care chemotherapy. Note for (**b**): The upper triangle of the matrix displays only the pooled effect sizes of the direct comparisons available in the network. Fields that remain empty in the upper triangle mean that the direct evidence for the comparison is not available. The lower triangle shows the estimated effect sizes for each comparison even when only indirect evidence is available. In the lower triangle of the matrix, values in each cell represent the hazard ratio (HR and 95% confidence interval) of the intervention at the top, compared to the comparator on the left. When HR < 1, prefers the column intervention, indicating that the column intervention is more effective than the row intervention on reducing overall survival. When HR > 1, prefers the row intervention. Significant results are in bold and underlined for both upper and lower triangles. (**c**) Network plot of comparisons among different anti-angiogenic agents and standard of care chemotherapy for progression-free survival in the platinum-sensitive setting. Key for (**c**): *Bev_C* Bevacizumab (concurrent), *Bev_CM* Bevacizumab (concurrent + maintenance), *Ced_CM* Cediranib (concurrent + maintenance), *Chemo* Standard of care chemotherapy. Note for (**c**): Nodes represent the interventions, lines connecting nodes represent direct comparisons between pairs of interventions, the number stated on the lines represents the number of studies involved in the comparisons. (**d**) Comparative effectiveness of different anti-angiogenic agents and standard of care chemotherapy for progression-free survival in the platinum-sensitive setting. Key for (**d**): *Bev_C* Bevacizumab (concurrent), *Bev_CM* Bevacizumab (concurrent + maintenance), *Ced_CM* Cediranib (concurrent + maintenance); Chemo, Standard of care chemotherapy. Note for (**d**): The upper triangle of the matrix displays only the pooled effect sizes of the direct comparisons available in the network. Fields that remain empty in the upper triangle mean that the direct evidence for the comparison is not available. The lower triangle shows the estimated effect sizes for each comparison even when only indirect evidence is available. In the lower triangle of the matrix, values in each cell represent the hazard ratio (HR and 95% confidence interval) of the intervention at the top, compared to the comparator on the left. When HR < 1, prefers the column intervention, indicating that the column intervention is more effective than the row intervention on reducing overall survival. When HR > 1, prefers the row intervention. Significant results are in bold and underlined for both upper and lower triangles. (**e**) Comparative effectiveness of different anti-angiogenic agents and standard of care chemotherapy in platinum-sensitive setting: P-scores for overall survival. Key for (**e**): *Bev_C* Bevacizumab (concurrent), *Bev_CM* Bevacizumab (concurrent + maintenance), *Ced_CM* Cediranib (concurrent + maintenance), *Chemo* Standard of care chemotherapy. Notes for (**e**): The higher the P-scores, the higher likelihood that an intervention is in the top rank or one of the top ranks. (**f**) Comparative effectiveness of different anti-angiogenic agents and standard of care chemotherapy in platinum-sensitive setting: P-scores for progression-free survival. Key for (**f**): *Bev_C* Bevacizumab (concurrent), *Bev_CM* Bevacizumab (concurrent + maintenance), *Ced_CM* Cediranib (concurrent + maintenance); Chemo, Standard of care chemotherapy. Notes for (**f**): The higher the P-scores, the higher likelihood that an intervention is in the top rank or one of the top ranks.

There were four RCTs in the platinum-sensitive setting. Sensitivity analysis of the NMA results showed no significant difference in OS among the anti-angiogenic agents (Fig. 5a,b). Nonetheless, the combination of chemotherapy and maintenance treatment with (i) bevacizumab (Bev_CM) and (ii) cediranib (Ced_CM), as well as (iii) bevacizumab combined with chemotherapy alone (Bev_C) were significantly more effective than chemotherapy in improving PFS, with HRs of 0.48 (95% CI 0.36; 0.66), 0.56 (95% CI 0.41; 0.77) and 0.58 (95% CI 0.47; 0.70) respectively (Fig. 5c,d). Amongst these interventions, bevacizumab combined with chemotherapy and maintenance treatment (Bev_CM) had the highest probability of being the best option for prolonging PFS (p-score: 0.85). Effectiveness hierarchy ranking results of the interventions for OS and PFS are shown in Fig. 5e and f, respectively.

### Publication bias assessment

Based on the visual inspection of comparison-adjusted funnel plot of 15 included RCTs on the primary outcome of OS in the recurrent EOC setting, there was no evidence of funnel plot asymmetry (Egger's test: p = 0.8347, Supplementary Fig. 3), indicating absence of publication bias. However, for other NMAs on the OS outcome in the chemotherapy naïve setting, publication bias could not be assessed due to an insufficient number of included studies.

## Discussion

To our knowledge, this is the most comprehensive NMA which explores the clinical impact of anti-angiogenic agents in EOC. We demonstrate the probable lack of efficacy of anti-angiogenic agents in non-high-risk chemotherapy naïve and platinum-sensitive EOC. Furthermore, in the absence of a predictive biomarker for response to anti-angiogenic agents, we highlight their probable positioning in managing high-risk chemotherapy naïve and platinum-resistant EOC.

The variation in OS outcomes reported in the high-risk chemotherapy naïve disease setting may make it difficult to infer any recommendations regarding the role of anti-angiogenic agents in this disease setting. The ICON7 trial demonstrated that the use of bevacizumab in high-risk disease is associated with an OS benefit (p = 0.01, HR 0.78; 95% CI 0.63–0.97)^57^. Conversely, the AGO-OVAR 12 study reported that OS in the high-risk group did not favor nintedanib (HR: 1.14; 95% CI 0.·89–1.45)^56^. Additionally, the GOG-0218 trial demonstrated that FIGO stage IV disease favored the concurrent use of bevacizumab with chemotherapy followed by bevacizumab maintenance arm (HR: 0.72; 95% CI 0.53–0.97)^58^. When used concurrently with chemotherapy followed by maintenance until progression, we demonstrate that bevacizumab was associated with the highest probability of OS (Fig. 2b) and PFS (Fig. 2d) benefit in the high-risk chemotherapy naïve EOC setting. Furthermore, we demonstrate that anti-angiogenic agents may not play a role in managing non-high-risk chemotherapy naïve EOC.

Interestingly, platinum resistance was associated with a significant PFS and OS benefit from the addition of anti-angiogenic agents to chemotherapy. In addition, we identified that in the setting of platinum-resistant disease, Pazopanib (P-score = 0·79) and Sorafenib (P-score = 0·76), administered concurrently with chemotherapy, resulted in abclinically significant improvement in OS (Fig. 4).

The FDA defines clinical outcomes as a direct measure of benefit from an intervention in a trial. A surrogate endpoint is used as a predictive substitute for clinical benefit. Nevertheless, surrogate outcome measures play a notable role in solid cancers^59^. PFS is the most commonly used primary endpoint and surrogate marker for OS in solid cancer^60,61^, and its use is growing. The FDA has approved many cancer drugs based on surrogate endpoint data. Among those receiving regulatory approval, 57% of cancer drugs did not demonstrate an OS benefit^62^.

Pasalic et al. and Prasad et al. highlight the suboptimal predictive value of PFS as a surrogate for OS^63,64^. Additionally, in EOC, the predictive value of PFS was further refuted as a surrogate for OS^65^. Furthermore, interpretation of the result findings presented in this NMA show that PFS falls short of being a substitute for OS in EOC. Therefore, we consider that the findings presented in this NMA are collectively significant and highlight the importance of selecting the appropriate EOC patient group, high-risk and platinum-resistant disease, that are likely to derive OS benefit from anti-angiogenic agents. Consequently, regulators need to cautiously interpret trials that report PFS benefits without mature OS data.

To date, there are no clinically approved predictive molecular biomarkers that may have clinical utility in selecting the subgroup of patients likely to benefit from anti-angiogenic agents. However, there is a clear clinical unmet need. Nevertheless, there has been a significant drive to identify patients who benefit from bevacizumab. Bentink et al. identified molecular subtypes (split 1–4) in high-grade serous ovarian cancer (HGSOC, generated from 129 formalin fixed parrafin imbedded (FFPE) patient tumor samples. Split 1 was associated with angiogenesis and extracellular matrix proteins, termed the angiogenic subtype, corresponding with the Tothill C1 and TCGA mesenchymal subtypes^66^.

Backen et al. identified that high Ang1/low Tie2 serum values were associated with significantly improved PFS in the ICON7 bevacizumab-treated patient cohort (median, 23.0 months vs. 16.2; p = 0.003)^67^. In addition, Gourley et al. developed a gene signature to identify angiogenic molecular subtypes in ovarian cancer^68^. These findings were validated in the ICON7 trial and demonstrated that bevacizumab had a favorable PFS impact on patient tumors harboring the angiogenic subtype^68^.

Collinson et al. identified three potentially predictive biomarkers, mesothelin, fms-like tyrosine kinase-4 (*FLT4*), and α_1_-acid glycoprotein (*AGP*), from serum samples of patients recruited to the ICON7 trial. These biomarkers identified the subgroup of patients likely to benefit from bevacizumab, predominantly leading to an improvement in median PFS of 5.5 months in the signature positive subgroup (p = 0.001)^69^. In the GOG218 trial, Birrer et al. demonstrated a correlation between microvessel density (MVD), tumor VEGF-A (tVEGF-A) expression, and survival outcome in the bevacizumab arm. Comparing chemotherapy with bevacizumab versus chemotherapy alone, higher MVD showed predictive value for PFS (p = 0.018) and OS (p = 0.0069). tVEGF-A expression showed potential predictive value for OS (p = 0.023)^70^. However, these biomarker findings need to be independently validated in more extensive trials before being implemented into clinical practice.

We identified the strengths and limitations of this study. This study helped clarify the survival outcomes of multiple anti-angiogenic agent comparators in different disease settings in EOC, thereby creating a hierarchical overview of best to worst treatment regimens. Furthermore, this study provided a comprehensive insight into the potential impact of anti-angiogenic agents in EOC by estimating their effect using both direct and indirect comparisons.

However, It is essential to highlight that the small sample size in the MITO-11 trial^71^ and the TRIAS trial^72^ may impact the interpretation of the OS results illustrated in Fig. 4. Therefore, making it challenging to definitively assess the impact of pazopanib on OS in platinum-resistant disease. In addition, not all the trials identified in the recurrent disease setting reported separate PFS and OS based on PFI. This, therefore, reduced the number of trials included in the sensitivity analysis for each pre-defined PFI cohort. Finally, this systematic review and NMA aimed to synthesize evidence of anti-angiogenic agents' comparative effectiveness for improving OS among EOC patients. However, we did not perform network meta-regression to explore the between-study heterogeneity variance, which may be a future research recommendation.

Following our observations, we suggest a potential treatment approach (Supplementary Fig. 4) for EOC. In high-risk treatment naïve disease, bevacizumab combined with standard chemotherapy and followed bevacizumab maintenance may provide the best PFS and OS outcomes. On recurrence, anti-angiogenic agents, particularly pazopanib in combination with chemotherapy, may be the most appropriate in managing platinum-resistant EOC. In contrast, PARP inhibitors combined with anti-angiogenic agents may play a more critical role than anti-angiogenic agent monotherapy in platinum-sensitive EOC^73^. The level IA evidence presented in this study further emphasizes the need for better patient selection for anti-angiogenic agents in EOC.

## Supplementary Information

Supplementary Information 1.

Supplementary Information 2.

Supplementary Information 3.

## References

[1] World Health Organization. Globocan 2020. *Int. Agency Res.* (2020).

[2] Pujade-Lauraine E (2014). Bevacizumab combined with chemotherapy for platinum-resistant recurrent ovarian cancer: The AURELIA open-label randomized phase III trial. J. Clin. Oncol..

[3] Aghajanian C (2012). OCEANS: A randomized, double-blind, placebo-controlled phase III trial of chemotherapy with or without bevacizumab in patients with platinum-sensitive recurrent epithelial ovarian, primary peritoneal, or fallopian tube cancer. J. Clin. Oncol..

[4] Coleman RL (2016). Bevacizumab after bevacizumab in platinum-sensitive recurrent ovarian cancer: A subgroup analysis of GOG0213. J. Clin. Oncol..

[5] Ledermann JA (2013). Randomised double-blind phase III trial of cediranib (AZD 2171) in relapsed platinum sensitive ovarian cancer: Results of the ICON6 trial. Eur. J. Cancer.

[6] Ledermann J (2012). Olaparib maintenance therapy in platinum-sensitive relapsed ovarian cancer. N. Engl. J. Med..

[7] Wilson MK (2016). 5th ovarian cancer consensus conference of the gynecologic cancer intergroup: Recurrent disease. Ann. Oncol..

[8] Friedlander M (2011). Clinical trials in recurrent ovarian cancer. Int. J. Gynecol. Cancer.

[9] Cannistra SA (2004). Cancer of the ovary. N. Engl. J. Med..

[10] Naumann RW, Coleman RL (2011). Management strategies for recurrent platinum-resistant ovarian cancer. Drugs.

[11] Folkman J, Folkman J (2007). Angiogenesis: An organizing principle for drug discovery?. Nat. Rev. Drug Discov..

[12] Oklu R, Walker TG, Wicky S, Hesketh R (2010). Angiogenesis and current anti-angiogenic strategies for the treatment of cancer. J. Vasc. Interv. Radiol..

[13] Folkman J (1971). Tumor angiogenesis: Therapeutic implications. N. Engl. J. Med..

[14] Bergers G, Benjamin LE (2003). Tumorigenesis and the angiogenic switch. Nat. Rev. Cancer.

[15] Carmeliet P, Jain RK (2011). Molecular mechanisms and clinical applications of angiogenesis. Nature.

[16] Hanahan D (2022). Hallmarks of cancer: New dimensions. Cancer Discov..

[17] Coleman RL (2017). Bevacizumab and paclitaxel-carboplatin chemotherapy and secondary cytoreduction in recurrent, platinum-sensitive ovarian cancer (NRG Oncology/Gynecologic Oncology Group study GOG-0213): A multicentre, open-label, randomised, phase 3 trial. Lancet Oncol..

[18] Perren TJ (2011). A phase 3 trial of bevacizumab in ovarian cancer. N. Engl. J. Med..

[19] Burger RA (2011). GOG 218: Incorporation of bevacizumab in the primary treatment of ovarian cancer. N. Engl. J. Med..

[20] Cancer, O. *NCCN.org NCCN Guidelines for Patients *®. www.nccn.org/patients (2021).

[21] Colombo N (2019). ESMO-ESGO consensus conference recommendations on ovarian cancer: Pathology and molecular biology, early and advanced stages, borderline tumours and recurrent disease. Ann. Oncol..

[22] Managing advanced (stage II–IV) ovarian cancer: NICE Pathways. https://pathways.nice.org.uk/pathways/ovarian-cancer#path=view%3A/pathways/ovarian-cancer/managing-advanced-stage-ii-iv-ovarian-cancer.xml&content=view-node%3Anodes-second-line-and-subsequent-chemotherapy.

[23] Wang H, Xu T, Zheng L, Li G (2018). Angiogenesis inhibitors for the treatment of ovarian cancer: An updated systematic review and meta-analysis of randomized controlled trials. Int. J. Gynecol. Cancer.

[24] Wu YS, Shui L, Shen D, Chen X (2017). Bevacizumab combined with chemotherapy for ovarian cancer: An updated systematic review and meta-analysis of randomized controlled trials. Oncotarget.

[25] Marchetti C (2016). Efficacy and toxicity of bevacizumab in recurrent ovarian disease: An update meta-analysis on phase III trials. Oncotarget.

[26] Jiang Y, Sun X, Kong B, Jiang J (2018). Antiangiogenesis therapy in ovarian cancer patients: An updated meta-analysis for 15 randomized controlled trials. Medicine.

[27] Li J, Zhou L, Chen X, Ba Y (2015). Addition of bevacizumab to chemotherapy in patients with ovarian cancer: A systematic review and meta-analysis of randomized trials. Clin. Transl. Oncol..

[28] Zhou M, Yu P, Qu X, Liu Y, Zhang J (2013). Phase III trials of standard chemotherapy with or without bevacizumab for ovarian cancer: A meta-analysis. PLoS ONE.

[29] Ye Q, Chen HL (2013). Bevacizumab in the treatment of ovarian cancer: A meta-analysis from four phase III randomized controlled trials. Arch. Gynecol. Obstet..

[30] Li J, Li S, Chen R, Yu H, Lu X (2015). The prognostic significance of anti-angiogenesis therapy in ovarian cancer: A meta-analysis. J. Ovarian Res..

[31] Li X, Zhu S, Hong C, Cai H (2016). Angiogenesis inhibitors for patients with ovarian cancer: A meta-analysis of 12 randomized controlled trials. Curr. Med. Res. Opin..

[32] Wang TS (2014). A meta-analysis of bevacizumab combined with chemotherapy in the treatment of ovarian cancer. Indian J. Cancer.

[33] Hutton B (2015). The PRISMA extension statement for reporting of systematic reviews incorporating network meta-analyses of health care interventions: Checklist and explanations. Ann. Intern. Med..

[34] Booth A (2012). The nuts and bolts of PROSPERO: An international prospective register of systematic reviews. Syst. Rev..

[35] Higgins J, Savović J, Page MJ, Sterne JAC (2019). RoB 2: A revised Cochrane risk-of-bias tool for randomized trials. Br. Med. J..

[36] Lu G, Ades AE (2004). Combination of direct and indirect evidence in mixed treatment comparisons. Stat. Med..

[37] Mills EJ, Thorlund K, Ioannidis JPA (2013). Demystifying trial networks and network meta-analysis. BMJ.

[38] Neupane B, Richer D, Bonner AJ, Kibret T, Beyene J (2014). Network meta-analysis using R: A review of currently available automated packages. PLoS ONE.

[39] Rucker G, Krahn U, Konig J, Efthimiou O, Papakonstantinou T, Schwarzer G (2021). Package ‘netmeta’. Netw. Meta-Anal. Freq. Methods..

[40] Shim SR, Kim S-J, Lee J, Rücker G (2019). Network meta-analysis: Application and practice using R software. Epidemiol. Health.

[41] HarrerCuijpers P, Furukawa TA, Ebert DD (2021). Doing Meta-Analysis with R: A Hands-On Guide.

[42] White IR (2015). Network meta-analysis. Stata J..

[43] Ter Veer E, van Oijen MGH, van Laarhoven HWM (2019). The use of (network) meta-analysis in clinical oncology. Front. Oncol..

[44] Rücker G, Schwarzer G (2015). Ranking treatments in frequentist network meta-analysis works without resampling methods. BMC Med. Res. Methodol..

[45] Monk BJ, Minion LE, Coleman RL (2016). Anti-angiogenic agents in ovarian cancer: Past, present, and future. Ann. Oncol..

[46] Colomban O (2020). Bevacizumab for newly diagnosed ovarian cancers: Best candidates among high-risk disease patients (ICON-7). JNCI Cancer Spectr..

[47] Salanti G (2012). Indirect and mixed-treatment comparison, network, or multiple-treatments meta-analysis: Many names, many benefits, many concerns for the next generation evidence synthesis tool. Res. Synth. Methods.

[48] Salanti G, Del Giovane C, Chaimani A, Caldwell DM, Higgins JPT (2014). Evaluating the quality of evidence from a network meta-analysis. PLoS ONE.

[49] Higgins JPT, Thomas J, Chandler J, Cumpston M, Li T, Page MJ (2019). Cochrane Handbook for Systematic Reviews of Interventions.

[50] Veroniki AA, Vasiliadis HS, Higgins JPT, Salanti G (2013). Evaluation of inconsistency in networks of interventions. Int. J. Epidemiol..

[51] van Valkenhoef G, Dias S, Ades AE, Welton NJ (2016). Automated generation of node-splitting models for assessment of inconsistency in network meta-analysis. Res. Synth. Methods.

[52] Duska LR (2020). A randomized phase II evaluation of weekly gemcitabine plus pazopanib versus weekly gemcitabine alone in the treatment of persistent or recurrent epithelial ovarian, fallopian tube or primary peritoneal carcinoma. Gynecol. Oncol..

[53] Zang R (2013). Pazopanib (Paz) monotherapy in Asian women who have not progressed after first-line chemotherapy for advanced ovarian, Fallopian tube, or primary peritoneal carcinoma. J. Clin. Oncol..

[54] Vergote I (2019). Trebananib or placebo plus carboplatin and paclitaxel as first-line treatment for advanced ovarian cancer (TRINOVA-3/ENGOT-ov2/GOG-3001): A randomised, double-blind, phase 3 trial. Lancet Oncol..

[55] Coleman R (2013). Analysis of survivorship in high-risk patients on treated on GOG-218. Gynecol. Oncol..

[56] Ray-Coquard I (2020). Final results from GCIG/ENGOT/AGO-OVAR 12, a randomised placebo-controlled phase III trial of nintedanib combined with chemotherapy for newly diagnosed advanced ovarian cancer. Int. J. Cancer.

[57] Oza AM (2015). Standard chemotherapy with or without bevacizumab for women with newly diagnosed ovarian cancer (ICON7): Overall survival results of a phase 3 randomised trial. Lancet Oncol..

[58] Randall L (2013). Outcome differences in patients with advanced epithelial ovarian, primary peritoneal and fallopian tube cancers treated with and without bevacizumab. Gynecol. Oncol..

[59] Shi Q, Sargent DJ (2009). Meta-analysis for the evaluation of surrogate endpoints in cancer clinical trials. Int. J. Clin. Oncol..

[60] Kay A, Higgins J, Day AG, Meyer RM, Booth CM (2012). Randomized controlled trials in the era of molecular oncology: Methodology, biomarkers, and end points. Ann. Oncol..

[61] Davis S (2012). A Review of Studies Examining the Relationship between Progression-Free Survival and Overall Survival in Advanced or Metastatic Cancer.

[62] Kim C, Prasad V (2015). Cancer drugs approved on the basis of a surrogate end point and subsequent overall survival: An analysis of 5 years of us food and drug administration approvals. JAMA Intern. Med..

[63] Pasalic D (2020). Progression-free survival is a suboptimal predictor for overall survival among metastatic solid tumour clinical trials. Eur. J. Cancer.

[64] Prasad V, Kim C, Burotto M, Vandross A (2015). The strength of association between surrogate end points and survival in oncology: A systematic review of trial-level meta-analyses. JAMA Intern. Med..

[65] Paoletti X (2020). Assessment of progression-free survival as a surrogate end point of overall survival in first-line treatment of ovarian cancer: A systematic review and meta-analysis. JAMA Netw. Open.

[66] Bentink S (2012). Angiogenic mRNA and microRNA gene expression signature predicts a novel subtype of serous ovarian cancer. PLoS ONE.

[67] Backen A (2014). The combination of circulating Ang1 and Tie2 levels predicts progression-free survival advantage in bevacizumab-treated patients with ovarian cancer. Clin. Cancer Res..

[68] Gourley C (2014). Molecular subgroup of high-grade serous ovarian cancer (HGSOC) as a predictor of outcome following bevacizumab. J. Clin. Oncol..

[69] Collinson F (2013). Predicting response to bevacizumab in ovarian cancer: A panel of potential biomarkers informing treatment selection. Clin. Cancer Res..

[70] Birrer MJ (2015). Retrospective analysis of candidate predictive tumor biomarkers (BMs) for efficacy in the GOG-0218 trial evaluating front-line carboplatin–paclitaxel (CP) ± bevacizumab (BEV) for epithelial ovarian cancer (EOC). J. Clin. Oncol..

[71] Pignata S (2015). Pazopanib plus weekly paclitaxel versus weekly paclitaxel alone for platinum-resistant or platinum-refractory advanced ovarian cancer (MITO 11): A randomised, open-label, phase 2 trial. Lancet Oncol..

[72] Chekerov R (2018). Sorafenib plus topotecan versus placebo plus topotecan for platinum-resistant ovarian cancer (TRIAS): A multicentre, randomised, double-blind, placebo-controlled, phase 2 trial. Lancet Oncol..

[73] Ray-Coquard I (2019). Olaparib plus bevacizumab as first-line maintenance in ovarian cancer. N. Engl. J. Med..

