# Supplementary material for: A comprehensive systematic review and network meta-analysis: the role of anti-angiogenic agents in advanced epithelial ovarian cancer
Source: Sci Rep. 2022 Mar 9;12:3803. doi: 10.1038/s41598-022-07731-1 (PMC8907284; doi:10.1038/s41598-022-07731-1)
Supplement: Supplementary file 1 — Supplementary Information 1. [file 41598_2022_7731_MOESM1_ESM.docx]

Supplementary Fig 1: PRISMA flowchart of literature search and selection for randomized controlled trials

**Studies included in quantitative synthesis (network meta-analysis)**
**(n = 23)**

**Studies included in qualitative synthesis**
(n = 23)

**Articles excluded, with reasons
(n = 148):**

Abstracts (n= 65)

Biomarker Studies (n= 10)

Cost effectiveness Studies (n= 3)

Non-Randomized Studies (n= 29)

Case Reports (n= 1)

Duplicates (n= 9)

Quality of Life Studies (n= 5)

Toxicity Studies (n= 2)

Updated Follow-up Studies (n= 13)

Exploratory Analysis (n= 2)

Non-Standard comparator arm (n= 6)

No Survival Outcome measure (n= 1)

No Hazard Ratio (n= 2)

**Full-text articles assessed for eligibility**
(n = 171)

**Additional records identified through other sources:**

Cochrane Central Register of Controlled Trials (CCTR)

Cochrane Database of Systematic Reviews (CDSR)

ClinicalTrials.gov

ASCO, and ESMO abstract database
(n = 201)

**Records identified through database searching:**

Embase

PubMed

OVID Medline
(n = 2162)

**Records excluded**
(n = 1,392)

**Records screened**
(n = 171)

**Records after duplicates removed**
(n = 1563)

**Identification**

**Eligibility**

**Included**

**Screening**

**Supplementary Fig 2. Comparative effectiveness of different anti-angiogenic agents and standard of care chemotherapy (i.e., Carboplatin/Paclitaxel) in chemotherapy naïve setting:**

1. **P-scores for overall survival**


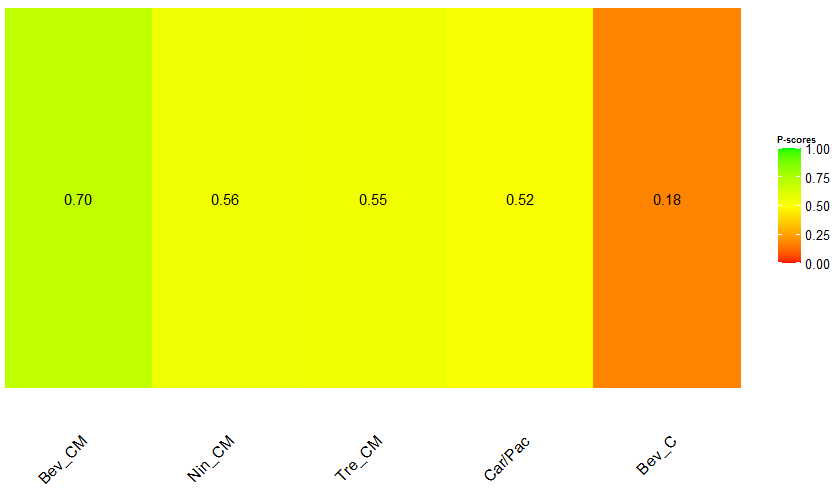


1. **P-scores for progression-free survival**


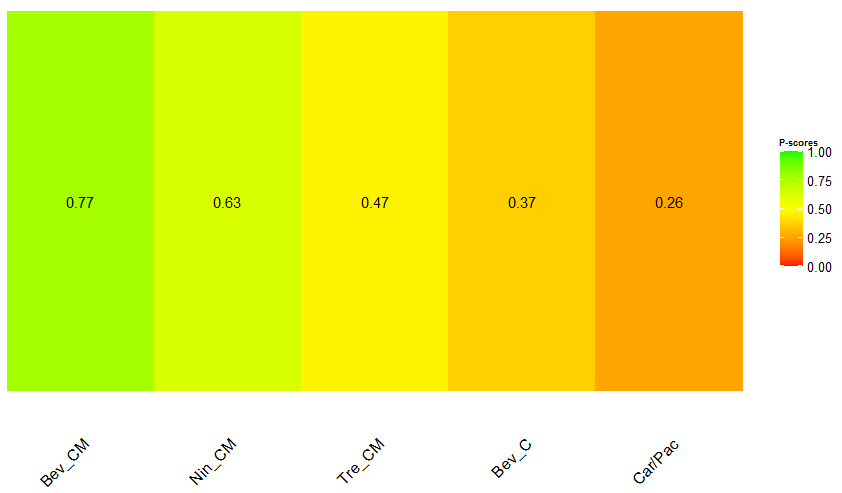


Key: Bev_C, Bevacizumab (concurrent); Bev_CM, Bevacizumab (concurrent + maintanence); Car/Pac, Carboplatin/Paclitaxel; Nin_CM, Nintedanib (concurrent + maintanence); Tre_CM, Trebananib (concurrent + maintanence)

Note: The higher the P-scores, the higher likelihood that an intervention is in the top rank or one of the top ranks.

**Supplementary Fig 3. Comparison-adjusted funnel plot of 15 included RCTs on the primary outcome of OS in recurrent EOC setting**


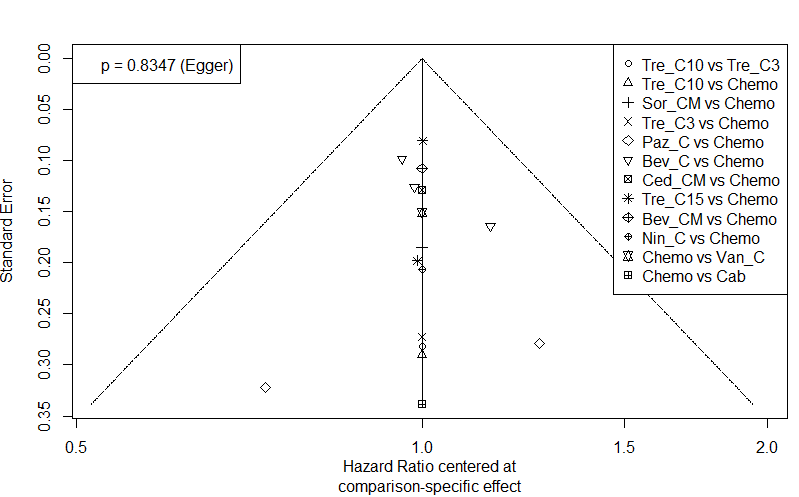


Key: Bev_C, Bevacizumab (concurrent); Bev_CM, Bevacizumab (concurrent + maintenance); Cab, Cabozantinib; Ced_CM, Cediranib (concurrent + maintenance); Chemo, Standard of care chemotherapy; Nin_C, Nintedanib (concurrent); Paz_C, Pazopanib (concurrent); Sor_CM, Sorafenib (concurrent + maintenance); Tre_C3, Trebananib (3mg/kg) (concurrent); Tre_C10, Trebananib (10mg/kg) (concurrent); Tre_C15, Trebananib (15mg/kg) (concurrent); Van_C, Vandetanib (concurrent).

**Supplementary Fig 4. Flow diagram to summarize the recommendations from the observations in this network meta-analysis**


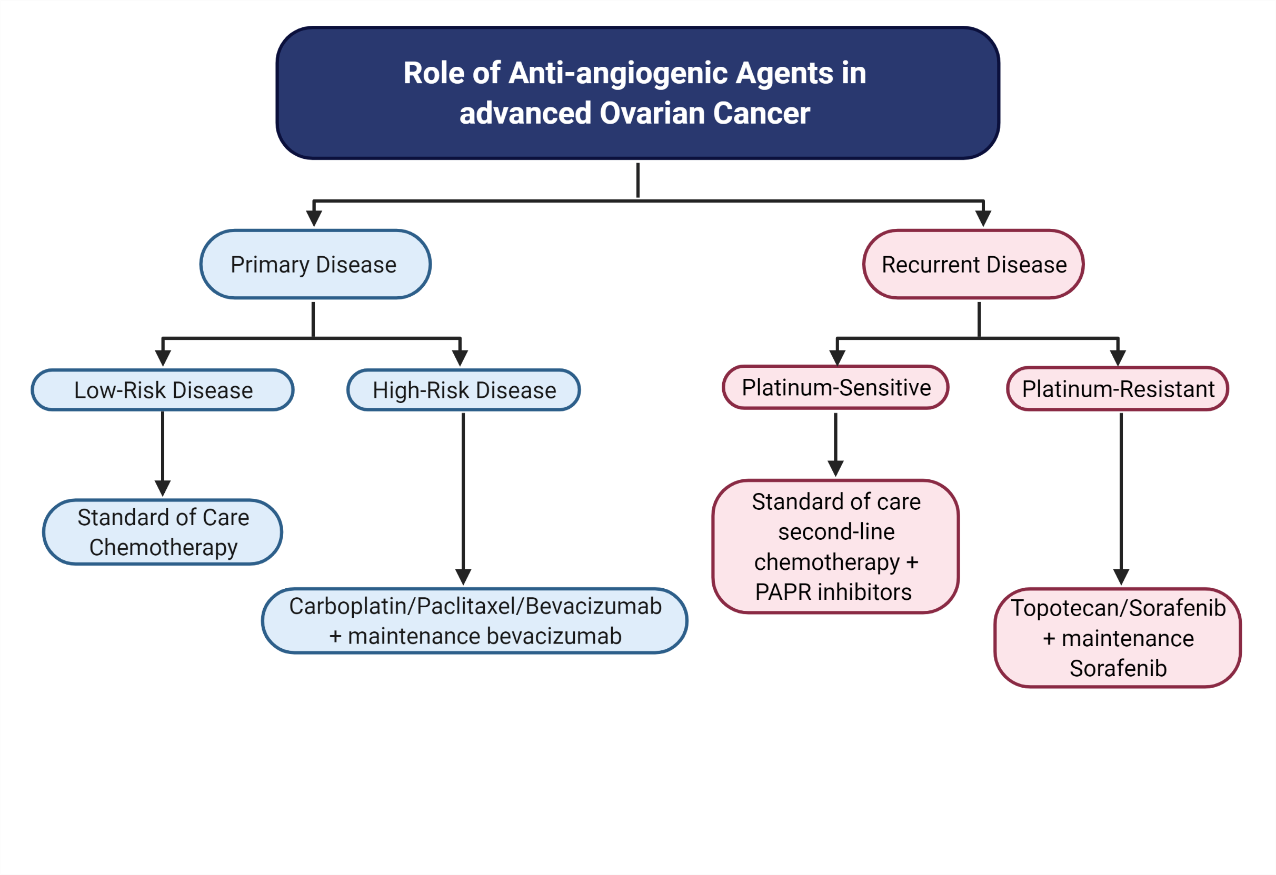


Table 1: The main characteristics of trials included in the network meta-analysis

| Trial_name | Phase | First Author | Journal | Year of publication | Disease Setting | Control Arm | Treatment Arm | No. of patients in Control Arm | No. of patients in Treatment Arm | Overall Survival (HR) | Overall Survival (95% CI) | Overall Survival Median (Control Arm, Months) | Overall Survival Median (Treatment Arm, Months) | Progression Free-Survival (HR) | Progression Free-Survival (95% CI) | Progression Free-Survival Median (Control Arm, Months) | Progression Free-Survivall Median (Treatment Arm, Months) | DOI |
| --- | --- | --- | --- | --- | --- | --- | --- | --- | --- | --- | --- | --- | --- | --- | --- | --- | --- | --- |
| AGO-OVAR 12 | III | Ray-Coquard et al | Int J Cancer | 2020 | Chemotherapy naïve | Standard of care chemotherapy - Carboplatin/Paclitaxel | Nintedanib (concurrent + maintanence) | 455 | 911 | 0.99 | 0.83-1.17 | 62.8 | 62.0 | 0.86 | 0.75-0.98 | 16.6 | 17.6 | 10.1002/ijc.32606 |
| ICON7 | III | Oza et al | Lancet Oncol | 2015 | Chemotherapy naïve | Standard of care chemotherapy - Carboplatin/Paclitaxel | Bevacizumab (concurrent + maintanence) | 764 | 764 | 0.99 | 0.85-1.14 | 58.6 | 58.0 | 0.93 | 0.83-1.05 | 17.5 | 19.9 | 0.1016/s1470-2045(15)00086-8 |
| GOG-0218 | III | Tewari et al | JCO | 2019 | Chemotherapy naïve | Standard of care chemotherapy - Carboplatin/Paclitaxel | Bevacizumab (concurrent + maintanence) | 625 | 623 | 0.96 | 0.85-1.09 | 41.1 | 43.4 | 0.717 | 0.625-0.824 | 10.3 | 14.1 | 10.1200/jco.19.01009 |
|  |  |  |  |  |  | Standard of care chemotherapy - Carboplatin/Paclitaxel | Bevacizumab (concurrent) |  | 625 | 1.06 | 0.94-1.2 | 41.1 | 40.8 | 0.908 | 0.795-1.04 | 10.3 | 11.2 | 10.1200/jco.19.01009 |
| TRINOVA-3 | III | Vergote et al | Lancet Oncol | 2019 | Chemotherapy naïve | Standard of care chemotherapy - Carboplatin/Paclitaxel | Trebananib (concurrent + maintanence) | 337 | 678 | 0.99 | 0.79-1.25 | 43.6 | 46.6 | 0.93 | 0.79-1.09 | 15.0 | 15.9 | 10.1016/s1470-2045(19)30178-0 |
| Herzog et al | II | Herzog et al | Gynecol Onc | 2013 | First-line maintanence | Placebo | Sorafenib (maintenance) | 123 | 123 | 1.49 | 0.69-3.23 | NR | NR | 1.09 | 0.72-1.63 | 15.7 | 12.7 | 10.1016/j.ygyno.2013.04.011 |
| East Asian Study | II | Kim et al | Int J Gynecol Cancer | 2018 | First-line maintanence | Placebo | Pazopanib (maintenance) | 72 | 73 | NR | NR | NR | NR | 0.984 | 0.595-1.626 | 18.1 | 18.1 | 10.1097/igc.0000000000000602 |
| AGO-OVAR16 | III | Vergote et al | Gynecol Onc | 2019 | First-line maintanence | Placebo | Pazopanib (maintenance) | 468 | 472 | 0.96 | 0.805-1.145 | 64.0 | 59.1 | 0.802 | 0.678-0.949 | NR | NR | 10.1016/j.ygyno.2019.08.024 |
| AURELIA | III | Pujade-Lauraine et al | JCO | 2014 | Recurrent disease- Platinum resistant | Standard of care chemotherapy | Bevacizumab (concurrent) | 182 | 179 | 0.85 | 0.66-1.08 | 13.3 | 16.6 | 0.48 | 0.38-0.6 | 3.4 | 6.7 | 10.1200/jco.2013.51.4489 |
| TRIAS | II | Chekerov et al | Lancet Oncol | 2018 | Recurrent disease- Platinum resistant | Standard of care chemotherapy | Sorafenib (concurrent + maintanence) | 89 | 83 | 0.65 | 0.45-0.93 | 10.1 | 17.1 | 0.60 | 0.43-0.83 | 4.4 | 6.7 | 10.1016/s1470-2045(18)30372-3 |
| MITO 11 | II | Pignata et al | Lancet Oncol | 2015 | Recurrent disease- Platinum resistant | Standard of care chemotherapy | Pazopanib (concurrent) | 36 | 37 | 0.6 | 0.32-1.13 | 13.7 | 19.1 | 0.42 | 0.25-0.69 | 3.49 | 6.35 | 10.1016/s1470-2045(15)70115-4 |
| GOG-0213 | III | Coleman et al | Lancet Oncol | 2017 | Recurrent disease- Platinum sensitive | Standard of care chemotherapy | Bevacizumab (concurrent) | 337 | 337 | 0.829 | 0.683-1.005 | 37.3 | 42.2 | 0.628 | 0.534-0.739 | 10.4 | 13.8 | 10.1016/s1470-2045(17)30279-6 |
| MITO16b/MANGO–OV2/ENGOT–ov17 | III | Pignata et al | Lancet Oncol | 2021 | Recurrent disease- Platinum sensitive | Standard of care chemotherapy | Bevacizumab (concurrent) | 203 | 203 | 0.99 | 0.73-1.39 | 27.1 | 26.7 | 0.51 | 0.41-0.65 | 8.8 | 11.8 | 10.1016/S1470-2045(20)30637-9 |
| ICON6 | III | Ledermann et al | Lancet | 2016 | Recurrent disease- Platinum sensitive | Standard of care chemotherapy | Cediranib (concurrent + maintanence) | 118 | 164 | 0.86 | 0.67-1.11 | NR | NR | 0.56 | 0.44-0.72 | 8.7 | 11.0 | 10.1016/s0140-6736(15)01167-8 |
| OCEANS | III | Aghajanian et al | Gynecol Oncol | 2015 | Recurrent disease- Platinum sensitive | Standard of care chemotherapy | Bevacizumab (concurrent + maintanence) | 242 | 242 | 0.952 | 0.771-1.176 | 32.9 | 33.6 | 0.484 | 0.388-0.605 | 8.4 | 12.4 | 10.1016/j.ygyno.2015.08.004 |
| SWOG S0904 | II | Coleman et al | Eur J Cancer | 2014 | Recurrent disease | Standard of care chemotherapy | Vandetanib (concurrent) | 66 | 63 | 1.25 | 0.93-1.68 | 18.0 | 14.0 | 0.99 | 0.69-1.42 | 3.5 | 3.0 | 10.1016/j.ejca.2014.03.005 |
| Duska et al | II | Duska et al | Gynecol Onc | 2020 | Recurrent disease | Standard of care chemotherapy | Pazopanib (concurrent) | 73 | 75 | NR | NR | NR | NR | 0.61 | 0.4-0.992 | 2.9 | 5.3 | 10.1016/j.ygyno.2019.10.014 |
| Hall et al | II | Hall et al | Gynecol Onc | 2020 | Recurrent disease | Standard of care chemotherapy | Nintedanib (concurrent) | 55 | 59 | 1.03 | 0.69-1.55 | 6.4 | 6.8 | 0.91 | 0.62-1.32 | 2.6 | 2.9 | 10.1016/j.ygyno.2020.09.048 |
| Karlan et al | II | Karlan et al | JCO | 2012 | Recurrent disease | Standard of care chemotherapy | Trebananib (10mg/kg) (concurrent) | 55 | 53 | 0.6 | 0.34-1.06 | 20.9 | 22.5 | 0.76 | 0.49-1.18 | 4.6 | 7.2 | 10.1200/JCO.2010.34.3178 |
|  |  |  |  |  | Recurrent disease | Standard of care chemotherapy | Trebananib (3mg/kg) (concurrent) |  | 53 | 0.77 | 0.45-1.31 | 20.9 | 20.4 | 0.75 | 0.48-1.17 | 4.6 | 5.7 | 10.1200/JCO.2010.34.3178 |
| ENGOT-ov-6/TRINOVA-2 | III | Marth et al | Eur J Cancer | 2017 | Recurrent disease | Standard of care chemotherapy | Trebananib (concurrent) | 109 | 114 | 0.94 | 0.64-1.39 | 17.0 | 19.4 | 0.92 | 0.68-1.24 | 7.2 | 7.6 | 10.1016/j.ejca.2016.09.004 |
| Matulonis et al | II | Matulonis et al | Gynecol Onc | 2019 | Recurrent disease | Standard of care chemotherapy | Cabozantinib | 54 | 57 | 2.27 | 1.17-4.41 | NR | 19.4 | 1.11 | 0.77-1.61 | 5.5 | 5.3 | 10.1016/j.ygyno.2018.12.008 |
| TRINOVA-1 | III | Monk et al | Gyne Oncol | 2016 | Recurrent disease | Standard of care chemotherapy | Trebananib (concurrent) | 458 | 461 | 0.95 | 0.81-1.11 | 18.3 | 19.3 | 0.70 | 0.61-0.80 | 5.4 | 7.4 | 10.1016/j.ygyno.2016.07.112 |
| Richardson et al | II | Richardson et al | JAMA Oncol | 2018 | Recurrent disease | Standard of care chemotherapy | Pazopanib (concurrent) | 52 | 54 | 1.04 | 0.6-1.79 | 23.3 | 20.7 | 0.84 | 0.57-1.22 | 6.2 | 7.5 | 10.1001/jamaoncol.2017.4218 |
| Ledermann et al | II | Ledermann et al | JCO | 2011 | Recurrent disease | Placebo | Nintedanib (maintenance) | 41 | 43 | 0.84 | 0.51-1.39 | NR | NR | 0.65 | 0.41-1.02 | NR | NR | 10.1200/jco.2010.33.5208 |

PFS: progression-free survival; OS: overall survival; HR: hazard ratio; CI: confidence interval; NR: not reported.

Supplementary Table 2: Systematic review demonstrating the outcome variabilities in meta-analyses performed in angiogenesis inhibitor randomized controlled trials over time.

| PMID | Authors | Journal/Book | Publication Year | Number of Clinical Trials included | Study population | Trial Outcome | PFS | OS | Front-line: PFS | Front-line: OS | High-risk: PFS | High-risk: OS | Recurrent Disease: PFS | Recurrent Disease: OS | Platinum-Sensitive Disease: PFS | Platinum-Sensitive Disease: OS | Platinum-Resistant Disease: PFS | Platinum-Resistant Disease: OS | DOI |
| --- | --- | --- | --- | --- | --- | --- | --- | --- | --- | --- | --- | --- | --- | --- | --- | --- | --- | --- | --- |
| 24324725 | Zhou M et al | PLoS One | 2013 | 4 | 2 trials treatment naïve; 1 trial platinum sensitive; 1 trial platinum resistant | PFS benefit in newly diagnosed and recurrent ovarian cancer. OS benefit in newly diagnosed ovarian cancer only |  |  | HR, 0.82; 95% CI, 0.75-0.89 | HR, 0.87; 95% CI, 0.77-0.99 |  |  | HR, 0.48; 95% CI, 0.41-0.57 |  |  |  |  |  | 10.1371/journal.pone.0081858 |
| 23543268 | Ye Q et al | Arch Gynecol Obstet | 2013 | 4 | 2 trials treatment naïve; 1 trial platinum sensitive; 1 trial platinum resistant | ORR and PFS benefit | HR, 0.691; 95% CI, 0.517-0.865 | HR, 0.934; 95% CI, 0.826-1.041 |  |  |  |  |  |  |  |  |  |  | 10.1007/s00404-013-2820-1 |
| 25818743 | Wang TS et al | Indian J Cancer | 2014 | 5 | 2 trials treatment naïve; 1 trial platinum sensitive; 1 trial recurrent disease; 1 breast cancer trial | PFS benefit | HR, 0.64; 95% CI, 0.46-0.82 | HR, 0.84; 95% CI, 0.59-10.9 |  |  |  |  |  |  |  |  |  |  | 10.4103/0019-509X.154084 |
| 25990506 | Li J et al | Clin Transl Oncol | 2015 | 4 | 2 trials treatment naïve; 1 trial platinum-sensitive; 1 trial platinum-resistant | PFS benefits in newly diagnosed and recurrent ovarian cancer. OS benefit in newly diagnosed ovarian cancer only |  |  | HR, 0.82; 95% CI 0.75-0.89 | HR, 0.86; 95% CI 0.75-0.99 |  |  | HR, 0.48; 95% CI 0.41-0.57 | HR, 0.93; 95% CI 0.78-1.12 |  |  |  |  | 10.1007/s12094-015-1293-z |
| 4526298 | Li J et al | J Ovarian Res | 2015 | 12 | 3 trials treatment naïve; 1 trial maintenance; 4 trials recurrent disease; 3 trials platinum sensitive; 1 trial platinum resistant | PFS and OS benefit | HR, 0.66; 95% CI, 0.58-0.75 | HR, 0.89; 95% CI, 0.82-0.97 |  |  |  |  |  |  |  |  |  |  | 10.1186/s13048-015-0181-z |
| 26657509 | Marchetti C et al | Oncotarget | 2016 | 3 | 2 trials platinum-sensitive; 1 trial platinum resistant | PFS and OS benefit in Recurrent Disease |  |  |  |  |  |  | HR, 0.53; 95% CI 0.44 − 0.63 | HR, 0.87; 95% CI, 0.77 to 0.99 |  |  |  |  | 10.18632/oncotarget.6507 |
| 26652645 | Li X et al | Curr Med Res Opin | 2016 | 12 | 4 trials treatment naïve; 2 trial maintenance; 3 trials recurrent disease; 2 trials platinum sensitive; 1 trial platinum resistant | PFS benefit | HR, 0.61; 95% CI, 0.48-0.79 |  |  |  |  |  |  |  |  |  |  |  | 10.1185/03007995.2015.1131152 |
| 27793044 | Wu YS et al | Oncotarget | 2017 | 5 | 2 trials treatment naïve; 2 trial platinum-sensitive; 1 trial platinum-resistant | PFS and OS benefit in High-Risk disease and Recurrent disease |  |  | HR, 0.85; 95%CI 0.70-1.02 | HR, 0.94; 95%CI 0.84-1.05 | HR, 0.76; 95%CI 0.68-0.84 | HR, 0.85; 95%CI 0.74-0.96 | HR, 0.53; 95%CI 0.45-0.63 | HR, 0.87; 95%CI 0.77-0.99 |  |  |  |  | 10.18632/oncotarget.12926 |
| 30142803 | Jiang Y et al | Medicine (Baltimore) | 2018 | 15 | 3 trials treatment naïve; 3 trials maintenance; 2 trials recurrent disease; 3 trials platinum-sensitive; 3 trials platinum resistant | PFS benefit in newly diagnosed and recurrent ovarian cancer. OS benefit in Recurrent disease |  |  | HR, 0.88; 95% CI 0.79-0.98 |  |  |  | HR, 0.58; 95% CI 0.52-0.65 | HR, 0.84; 95% CI 0.76-0.92 | HR, 0.56; 95% CI 0.48-0.64 | HR, 0.86; 95% CI 0.76-0.98 | HR, 0.54; 95% CI 0.41-0.71 | HR, 0.84; 95% CI 0.71-0.98 | 10.1097/MD.0000000000011920 |
| 29561301 | Wang H et al | Int J Gynecol Cancer | 2018 | 15 | 3 trials treatment naïve; 3 trials maintenance; 2 trials recurrent disease; 3 trials platinum-sensitive; 4 trials platinum-resistant | PFS and OS benefit in High-Risk disease and Recurrent disease |  |  | HR, 0.83; 95% CI, 0.71-0.97 | HR, 0.95; 95% CI, 0.86-1.05 | HR, 0.72; 95% CI, 0.65-0.81 | HR, 0.84; 95%CI, 0.74-0.96 | HR, 0.58; 95% CI, 0.52-0.65 | HR, 0.86; 95% CI, 0.79-0.94 |  |  |  |  | 10.1097/IGC.0000000000001258 |

PFS: progression-free survival; OS: overall survival; ORR: objective response rate; HR: hazard ratio; CI: confidence interval

Supplementary Table 3: Table to summarize the risk of bias assessment of trials included in the network meta-analysis

Supplementary Table 4: Risk of Bias assessment table

|  | **Randomization process** | **Deviations from intended interventions** | **Missing outcome data** | **Measurement of the outcome** | **Selection of the reported result** | **Overall Bias** |
| --- | --- | --- | --- | --- | --- | --- |
| Low risk | 91.3 | 78.3 | 100 | 65.2 | 100 | 52.2 |
| Some concerns | 8.7 | 21.7 | 0 | 34.8 | 0 | 47.8 |
| High risk | 0 | 0 | 0 | 0 | 0 | 0 |
